# Liouvillian of the Open STIRAP Problem

**DOI:** 10.3390/e20010020

**Published:** 2018-01-03

**Authors:** Thomas Mathisen, Jonas Larson

**Affiliations:** Department of Physics, Stockholm University, AlbaNova University Center, 106 91 Stockholm, Sweden

**Keywords:** STIRAP, Lindblad equation, quantum coherent control, exceptional points

## Abstract

With the corresponding Liouvillian as a starting point, we demonstrate two seemingly new phenomena of the STIRAP problem when subjected to irreversible losses. It is argued that both of these can be understood from an underlying Zeno effect, and in particular both can be viewed as if the environment assists the STIRAP population transfer. The first of these is found for relative strong dephasing, and, in the language of the Liouvillian, it is explained from the explicit form of the matrix generating the time-evolution; the coherence terms of the state decay off, which prohibits further population transfer. For pure dissipation, another Zeno effect is found, where the presence of a non-zero Liouvillian gap protects the system’s (adiabatic) state from non-adiabatic excitations. In contrast to full Zeno freezing of the evolution, which is often found in many problems without explicit time-dependence, here, the freezing takes place in the adiabatic basis such that the system still evolves but adiabatically.

## 1. Introduction

Coherent control has become an essential part of many branches in quantum physics, ranging from atomic and molecular thermal gases to solid state devises and ultracold atomic condensates [1,2,3,4]. Utilising adiabatic driving is a method to circumvent any errors arising from timing of applied pulses—as long as the process is adiabatic, the target state is reached. When a direct coupling between the initial and target state is forbidden by selection rules, an intermediate state is often used in a so called Raman coupling scheme. As often is the case, this additional state is an excited state that suffers from spontaneous emission. This source of dissipation/decoherence can be bypassed by using instead the so called STIRAP—stimulated Raman adiabatic passage method [1,5,6]. In addition, the STIRAP makes use of an intermediate state, but by properly choosing the two Raman coupling pulses, the coherent transfer of population can be made perfect without ever populating the excited intermediate state. As such, deficiencies due to spontaneous emission of that state are greatly suppressed [7,8]. However, dephasing of the two lower states may well occur, for example due to elastic collisions between particles [9]. The process is then no longer coherent, which indeed affects the population transfer. While the evolution is no longer unitary, it is still linear and generated by a Liouvillian operator. Naturally, the properties of the Liouvillian will determine the evolution of the system’s state.

In AMO experiments, the coupling to any environment can usually be made rather weak and especially when working in the optical regime the system dynamics can be well approximated by a Markovian master equation. The general form of such an equation is given by the Lindblad type [10]

(1) $$\partial_{t}\hat{\rho }=\hat{L}\left[\hat{\rho }\right]=i[\hat{\rho },\hat{H}]+D\left[\hat{\rho }\right],\;$$

with the disipator

(2) $$D\left[\hat{\rho }\right]=\sum_{i}\gamma_{i}\left(2{\hat{L}}_{i}\hat{\rho }{\hat{L}}_{i}^{†}-{\hat{L}}_{i}^{†}{\hat{L}}_{i}\hat{\rho }-\hat{\rho }{\hat{L}}_{i}^{†}{\hat{L}}_{i}\right).$$

Here, we have defined the Liouvillian $\hat{L},$ which generates the full evolution of the state, the unitary time-evolution governed by the system Hamiltonian $\hat{H}$, and the part incorporates the effects of the environment. The effective system–environment couplings are $\gamma_{i}$ with corresponding Lindblad jump operators ${\hat{L}}_{i}$. The Liouvillian on the form above preserves the physical properties of the state, that is $\hat{\rho }\left(t\right)$ is normalized and positive semi-definite for all times. That implies also that the evolution is in agreement with the fluctuation-dissipation theorem stating that any sort of dissipation inevitably causes some sort of decoherence. Neglecting such fluctuations, one of the first studies on the open STIRAP consisted of adding a complex component −iγ|2〉〈2| to the Hamiltonian [7]. In this simplified picture, it was found, via adiabatic elimination, that the transition probability $P_{i}$ for i=3 falls off exponentially in terms of the dissipation rate γ for weak dissipation $g_{0}\gg \gamma$, and as $\gamma^{-2}$ in the opposite limit $\gamma \gg g_{0}$. Ref. [8] considered instead the full Lindblad master Equation (1) describing spontaneous emission of the excited intermediate level. For a large regime of decay rates and being deep in the adiabatic regime, their results showed that the success rate was in principle unaltered by the openness of the problem. In a more recent work [11], the open STIRAP was reexamined by deriving a microscopic master equation that takes into account the explicit time-dependence of the Hamiltonian (In the standard derivation of the Lindblad master equation, the Hamiltonian is assumed time-independent. The derivation cannot be performed following the same procedure once the Hamiltonian becomes time-dependent.). In the overdamped regime, it was found that an increased population transfer is to be found, which results from a Zeno-type process. Dephasing between the two states |1〉 and |3〉 is expected to have a greater effect on the STIRAP process. In particular, dephasing among the lower states will effectively couple the different adiabatic states, and hence result in deterioration of the population transfer success rate. Influence of dephasing on the STIRAP problem has been analysed in Ref. [9] in the weak system–environment coupling limit.

In this work, instead of directly integrating the Lindblad master Equation (1), which has been the standard route in the past, we start from the properties of the Liouvillian $\hat{L}$ in order to analyse the general open STIRAP problem. We present new results that in a natural way can be explained in terms of the Liouvillian. In particular, by extracting the (complex) spectrum of $\hat{L}$, the different relevant time-scales can more easily be identified. For example, one may expect that, if the STIRAP process is slow, the environment will have a greater influence on the system dynamics, and possibly deteriorate the success rate. Simultaneously, a too fast STIRAP will imply non-adiabatic excitations taking you out from the desired instantaneous eigenstate. This expected behaviour is indeed found for weak couplings to the environment, and the optimal time for the process should be such that the inherent unitary and the external irreversible time scales agree. For strong dephasing, a surprising evolution is demonstrated. Here, it might actually be preferable to consider a slow process despite the coupling to the environment. It is explained from a Zeno freezing effect of the population transfer between the different diabatic states. Another Zeno manifestation is found for dissipation of the excited state. This is understood from the non-vanishing Liouvillian gap that tends to project the state back onto the desirable adiabatic state. There is a trade-off though, a too strong coupling to the environment implies a qualitatively different regime where the quantum Zeno mechanism projects you down to a diabatic state instead of the adiabatic state.

The paper is structured as follows. In the next section, we start by recapitulating the general idea behind STIRAP. The following subsection introduces the Bloch representation of the Lindblad master equation and we briefly discuss some needed properties of the Liouvillian matrix. We continue in Section 3 with the actual numerical results. In particular, the spectrum of the Liouvillian matrix is analysed in some detail. Furthermore, the full open STIRAP problem is simulated numerically, which confirms our predictions drawn from the structure of the Liouvillian matrix. Finally, we conclude in Section 4 with a summary and some remarks. In the Appendices, we especially point out some remarks about the Liouvillian matrix.

## 2. The Open STIRAP Model

The STIRAP model is a paradigm, both for demonstrating adiabatic ideas in general and as an example of a time-dependent three-level system. In this respect, it is a perfect starting point when analysing adiabatic processess from the perspective of the Liouvillian $\hat{L}\left(t\right)$. Indeed, the corresponding Liouvillian encapsulates many novel features, such as exceptional points (EP’s) [12,13]. The Liouvillians for open two-level systems, such as the Landau–Zener problem [14,15], seem to be lacking many of these interesting aspects.

### STIRAP for Closed Systems

**Figure 1:**
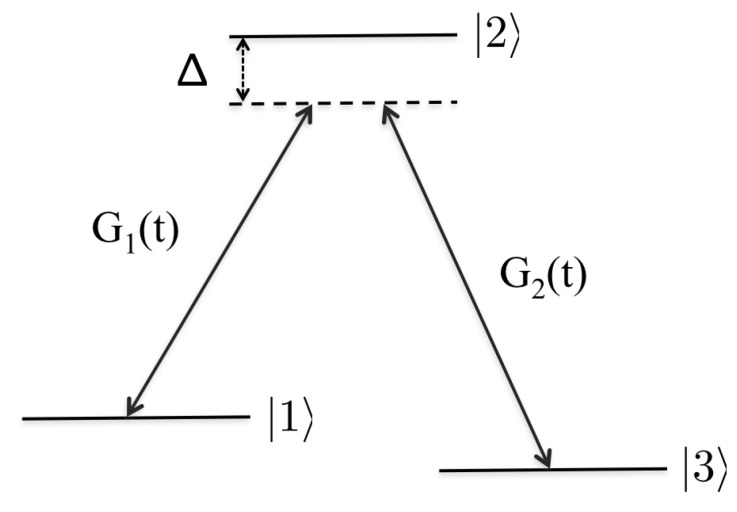
The traditional STIRAP Λ-scheme. Two pulses couple the lower stable states |1〉 and |3〉 with the excited state |2〉 with strengths $G_{1}\left(t\right)$ and $G_{2}\left(t\right)$. The lengths of the arrows symbolise the frequencies (in scaled units) of the light pulses, such that Δ marks the detuning between the applied pulses and the atomic transitions. In the figure, the two-photon transition is resonant such that only a single detuning parameter appears.

The standard STIRAP setup is the Λ one depicted in Figure 1; two stable states, |1〉 and |3〉 respectively, are laser coupled by a pump $G_{1}\left(t\right)$ and a Stokes field $G_{2}\left(t\right)$ to an excited intermediate state |2〉. Throughout, we assume a two-photon resonance transition, i.e., the photon frequency difference $\hbar (\omega_{2}-\omega_{1})$ (with $\omega_{1,2}$ the frequency of the pump and Stoke lasers respectively) matches the energy difference between the bare states |1〉 and |3〉. For the main part of this analytical subsection, we do not restrict the analysis to single photon resonance transitions, and thereby the introduction of a detuning Δ [1]. It is found, however, that the qualitative results will not depend on Δ and, for all numerical simulations, we thereby let Δ=0 for simplicity (see, for example, Ref. [16]). Before analysing the corresponding Liouvillian, we next summarise the basics of the STIRAP mechanism.

Within the rotating wave approximation and using the bare (diabatic) basis introduced above, the Schrödinger equation becomes [1] (ℏ=1 throughout)

(3) $$i\partial_{t}|\Psi \left(t\right)\rangle ={\hat{H}}_{d}\left(t\right)|\Psi \left(t\right)\rangle =\;\left[\begin{array}{ccc} 0 & G_{1}\left(t\right) & 0\\ G_{1}\left(t\right) & \Delta & G_{2}\left(t\right)\\ 0 & G_{2}\left(t\right) & 0 \end{array}\right]\;|\Psi \left(t\right)\rangle .$$

This equation defines the time-dependent Hamiltonian ${\hat{H}}_{d}$, and furthermore we have assumed real couplings $G_{1,2}\left(t\right)$ without loss of generality. The *adiabatic basis* $\left\{|\varphi_{+}\left(t\right)\rangle ,\;|\varphi_{0}\left(t\right)\rangle ,\;|\varphi_{-}\left(t\right)\rangle \right\}$ is given by the instantaneous (adiabatic) eigenstates of ${\hat{H}}_{d}\left(t\right)$. Written as a unitary matrix given in the diabatic basis, these states are

(4) $$\hat{U}\left(t\right)=\left[\begin{array}{ccc} \sin\phi \sin\theta & \cos\theta & \cos\phi \sin\theta \\ \cos\phi & 0 & -\sin\theta \\ \sin\phi \cos\theta & -\sin\theta & \cos\phi \cos\theta \end{array}\right],$$

with the parametrisation $\tan\theta =G_{1}\left(t\right)/G_{2}\left(t\right)$, $\tan2\phi =2G_{0}\left(t\right)/\Delta$, and with $G_{0}^{2}\left(t\right)=G_{1}^{2}\left(t\right)+G_{2}^{2}\left(t\right)$. The corresponding instantaneous (adiabatic) eigenstates are $E_{\pm }\left(t\right)=\left(\Delta \pm \sqrt{\Delta^{2}+4G_{0}^{2}\left(t\right)}\right)/2$ and $E_{0}\left(t\right)=0$. Thus, we have ${\hat{H}}_{\mathrm{ad}}\left(t\right)\equiv \hat{U}{\hat{H}}_{d}\left(t\right){\hat{U}}^{-1}=\mathrm{diag}\left(E_{+}\left(t\right),\;E_{0}\left(t\right),\;E_{-}\left(t\right)\right)$, with ${\hat{H}}_{\mathrm{ad}}\left(t\right)$ the adiabatic Hamiltonian. The Schrödinger equation in the adiabatic basis reads

(5) $$i\partial_{t}|\Psi_{\mathrm{ad}}\left(t\right)\rangle =\left[{\hat{H}}_{\mathrm{ad}}\left(t\right)-i{\hat{U}}^{-1}\partial_{t}\hat{U}\right]|\Psi_{\mathrm{ad}}\left(t\right)\rangle .$$

The adiabatic approximation consists of dropping the “gauge potential” $\hat{A}=-i\hat{U}\partial_{t}{\hat{U}}^{-1}$ that comprises the non-adiabatic couplings [1,17,18]. Thus, within this approximation, the adiabatic states evolve as $|\varphi_{i}\left(t\right)\rangle \to \exp\left(-i\int_{0}^{t}E_{i}\left(t^{\prime }\right)dt^{\prime }\right)|\varphi_{i}\left(t\right)\rangle$ with i=±,0 (Here, we put $\hat{A}\equiv 0$, but it is noted that this gauge potential is responsible for the Berry phase when encircling a closed loop in parameter space). The adiabatic state (also referred to as dark state) $|\varphi_{0}\left(t\right)\rangle$ is particularly attractive for practical purposes as it does not contain the bare excited state |2〉, which is typically subject to spontaneous emission. Now, if the couplings are chosen

(6) $$\begin{array}{c} \lim_{t\to -\infty }\frac{G_{1}\left(t\right)}{G_{2}\left(t\right)}=0,\;\theta \to 0,\\ \lim_{t\to +\infty }\frac{G_{2}\left(t\right)}{G_{1}\left(t\right)}=0,\;\theta \to \frac{\pi }{2}, \end{array}$$

it follows that, provided that the evolution is adiabatic, the adiabatic dark state obeys

(7) $$|\varphi_{0}\left(t\right)\rangle =\left\{\begin{array}{ccc} |1\rangle , & \; & t=-\infty ,\\ |3\rangle , & \; & t=+\infty . \end{array}\right.$$

This defines the STIRAP in our Λ-configuration; if we prepare our state in |1〉 and adiabatically turn on the couplings according to Equation (6), we will steer the state into |3〉 without ever populating the mediating state |2〉. One simple choice of couplings obeying this condition is for two symmetric Gaussians:

(8) $$\begin{array}{c} G_{1}\left(t\right)=g_{0}\exp\left[-\frac{{\left(t-a\tau \right)}^{2}}{2{\left(a\sigma \right)}^{2}}\right],\\ G_{2}\left(t\right)=g_{0}\exp\left[-\frac{{\left(t+a\tau \right)}^{2}}{2{\left(a\sigma \right)}^{2}}\right]. \end{array}$$

**Figure 2:**
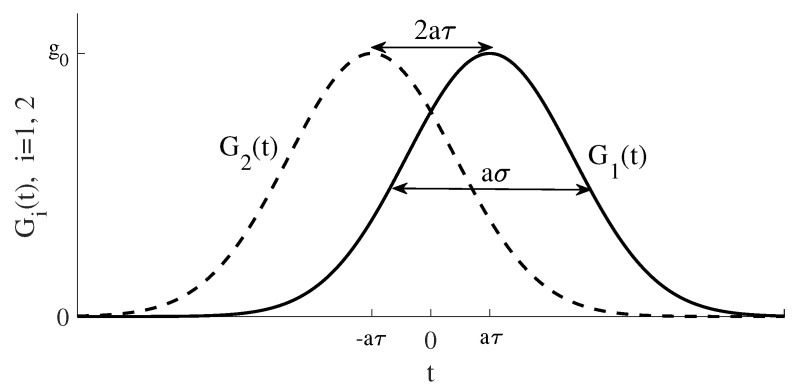
Counterintuitive STIRAP pulse sequenceof Equation (6). The pump pulse $G_{2}\left(t\right)$ is turned on before the Stokes pulse $G_{1}\left(t\right)$. The two pulse widths are aσ, the pulse separation 2aτ and the their amplitudes $g_{0}$. For this plot τ=σ which has been used for the numerics. Keeping the rest of the parameters fixed, for increasing adiabaticity parameter *a*, the two pulses become smoother, which favors a more adiabatic population transfer.

Here, $g_{0}$ is the pulse amplitude, aσ the pulse width, and 2aτ the distance between the pulses. These are shown in Figure 2, and they are also the pulses used throughout this manuscript. What is especially worth pointing out is the counterintuitive order of the pulses; pulse 2, the pump, which couples the initially empty states, is turned on before pulse 1, the Stokes. We have introduced *a* to serve as our single “adiabaticity parameter” (qualitatively, it is the pulse area setting the level of adiabaticity [5], and hence one could imagine varying other parameters, like the pulse amplitude, instead). Thus, we will keep $g_{0}$, τ and σ fixed in all numerical simulations (more precisely, $g_{0}=1$ and τ=σ=10) and instead vary *a* alone.

To get a better insight into the parameters rendering adiabatic evolution, we make use of the criteria for adiabaticity [18]

(9) $$\frac{\left|\langle \varphi_{+}\left(t\right)|(\partial_{t}{\hat{H}}_{d})|\varphi_{0}\left(t\right)\rangle \right|}{E_{+}^{2}\left(t\right)}\ll 1,$$

where we have used the symmetry of the $|\varphi_{\pm }\left(t\right)\rangle$ adiabatic states relative to $|\varphi_{0}\left(t\right)\rangle$, and that $E_{0}\left(t\right)=0$. This results in

(10) $$A\left(t\right)\equiv \frac{G_{1}\left(t\right)G_{2}\left(t\right)}{G_{0}^{3}\left(t\right)}\frac{a\tau }{{\left(a\sigma \right)}^{2}}\ll 1.$$

For adiabatic evolution, condition (10) should be fulfilled for all times $t\in [t_{i},t_{f}]$ between initial and final times. For not too large initial and final times, A(t) peaks at t=0, and we directly notice that since $A\left(t\right)∼1/g_{0}$ and A(t)∼1/a a large amplitude $g_{0}$ and/or a large *a* favour adiabaticity. As we already pointed out, we fix $g_{0}=1$ and instead vary *a* in order to analyse the influence of non-adiabatic excitations. As we will see, for the open STIRAP problem, *a* is in general not a good adiabaticity parameter.

## 3. Results and Discussion

There are in particular two types of dissipation/decoherence processes occurring naturally in realistic settings, spontaneous emission of the excited state |2〉 and dephasing. Previous works have considered direct numerical integration of the master equation or adiabatic elimination schemes [7,8,9]. Here, we examine the open STIRAP problem by rewriting the Lindblad master equation as a linear differential equation for the Bloch vector of the state. In two dimensions, the resulting equations are the famous Bloch equations [19]. In higher dimension, however, much less is known about these “generalised Bloch equations”, and the interpretation and analysis of the equations become also much more complex as will be discussed below.

The system evolution is modelled within the Lindblad master equation formalism, Equation (1). We will work in the diabatic basis such that the Hamiltonian is taken as ${\hat{H}}_{d}$ of Equation (3), and we consider two different situations of loss channels:

(1) Case (*a*). Dephasing of the lower states |1〉 and |3〉 implemented by the Lindblad jump operator
  (11) $$\hat{L}=\left|1\rangle \langle 1\right|-|3\rangle \langle 3|.$$
  We disregard any dephasing between the other levels as these are typically of less importance [9]. More precisely, once the dephasing arising from the jump operator (11) is taken into account, the additional dephasing occurring between the other levels do not qualitatively alter the results. This is especially true when the intermediate state is only slightly populated.
(2) Case (*b*). Spontaneous emission of the excited state |2〉 to the states |1〉 and |3〉. The corresponding jump operators are
  (12) $${\hat{L}}_{1}=|1\rangle \langle 2|,\;{\hat{L}}_{2}=|3\rangle \langle 2|.$$
  Note that we do not study the situation of losses to a fourth level |4〉 as was the scenario of Refs. [7,8]. Furthermore, we assume the same decay rates, $\kappa_{1}=\kappa_{2}=\kappa$.

**Figure 3:**
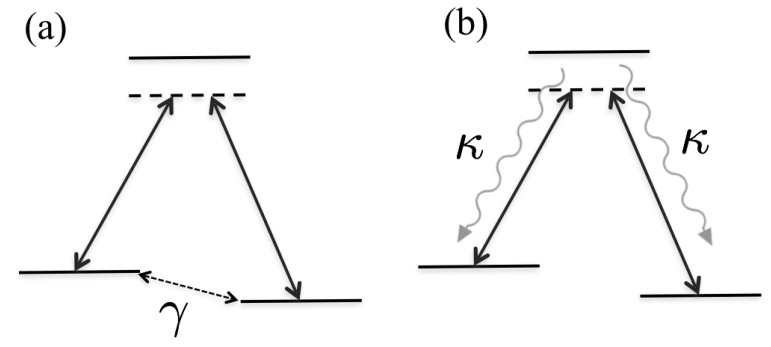
Schematic picture of the two open STIRAP situations: (**a**) dephasing of the two stable |1〉 and |3〉 states; and (**b**) spontaneous emission from the excited |2〉 state to either of the lower states.

These two cases are schematically presented in Figure 3.

### 3.1. The Liouvillian Matrix for the Λ System

Any state in any dimension *D* can be written on the form [20,21]

(13) $$\hat{\rho }=\frac{1}{D}\left(I+\sqrt{\frac{D(D-1)}{2}}\;R\cdot \lambda \right),$$

where $R=(r_{1},\;r_{2},\;\cdots ,\;r_{D^{2}-1})$ is the generalised *Bloch vector* and $\lambda =(\lambda_{1},\;\lambda_{2},\;\cdots ,\;\lambda_{D^{2}-1})$ a vector with the *generalised Gell–Mann matrices* as elements [22] (see Appendix A). For pure states, one has |R|=R=1, and naturally for the maximally mixed state R=0. Solving the master equation for $\hat{\rho }$ now transforms into the problem of solving an equation for the Bloch vector on the form [23,24,25]

(14) $$\partial_{t}R=MR+b.$$

Here, M is a $\left(D^{2}-1\right)\times \left(D^{2}-1\right)$ matrix generating the time-evolution of the Bloch vector, and will be hereafter called the Liouvillian matrix, while b we denote Liouvillian pump for reasons to be explained. In the Appendix B, we give several general properties of M. What is especially important is that it is not an Hermitian matrix, and thereby its eigenvalues $\mu_{i}$ are typically complex. The eigenvalues must, however, appear in complex conjugate pairs since the trace of M is real. Moreover, it follows that, whenever an eigenvalue is complex, the corresponding eigenvector must also be complex. As a result, since the Bloch vector is real for any physical state, most eigenvectors do actually not represent physical states $\hat{\rho }$. Another peculiarity, when dealing with non-Hermitian matrices, is that different eigenvectors need not be mutually orthogonal. In particular, at the EPs when two (or more) eigenvalues $\mu_{i}$ become degenerate the corresponding eigenvectors are parallel [12,13]. One quantity that is of special importance is the eigenvalue with the smallest (but non-zero) absolute value of its real part, i.e.,

(15) Δ˜=miniRe(−μi),

which sets an upper bound for the time-scale for reaching the steady state, and it is often referred to as the Liouvillian gap [26,27].

The Liouvillian matrices corresponding to the two cases (11) and (12), together with some of their properties, are presented in the Appendix C. As the couplings $G_{1}\left(t\right)$ and $G_{2}\left(t\right)$ now are time-dependent different scales become relevant. We will come back to this in the following subsection for the general case. In this subsection, however, we fix the values of the couplings, i.e., we consider the standard Raman Λ model. In particular, we consider $G_{1}=G_{2}=1$ and Δ=0. We have verified that varying these particular values does not alter our conclusions. With these parameters fixed, we ask how the eigenvalues of M vary with the system–environment couplings γ and κ. While keeping the couplings constant presents an oversimplified picture (loosely speaking assuming that the short time-scale is that of environment induced relaxation), it does give valuable insight. In this respect, this subsection is to be seen as providing an intuition for the dynamics given some non-Hermitian matrix M.

We may note that for γ=0 or κ=0, and since the Liouvillian matrices are skew-symmetric, we find two zero eigenvalues. The corresponding eigenstates are the steady states of the model. From the expression (A7) of the Appendix, it is easy to identify the Bloch vector

(16) $$R_{0}={\left[\begin{array}{cccccccc} 0 & 0 & \sqrt{3}c^{2} & -\sqrt{3}sc & 0 & 0 & 0 & \left(1-3s^{2}\right) \end{array}\right]}^{t}/2$$

as one of them, with s=sinθ and c=cosθ. This Bloch vector corresponds to the state with zero eigenvalue of the Raman model, which for the STIRAP system would be the adiabatic state $|\varphi_{0}\left(t\right)\rangle$ introduced in the previous section. As γ or κ become non-zero, the two zero eigenvalues split but stay real (and negative) [28]. The Bloch vector (16) is no longer representing a steady state. With a non-zero Liouvillian gap, one expects relaxation to the maximally mixed state, which would be the unique steady state. This is also what one finds for case (a) of dephasing. For case (b), the Liouvillian pump b is non-zero and this implies that the Bloch vector is still representing steady state. One may say that the pump counteracts the relaxation such that the steady state becomes a non-trivial state. This observation is important for understanding the STIRAP problem exposed to dissipation as we discuss in the following subsection.

**Figure 4:**
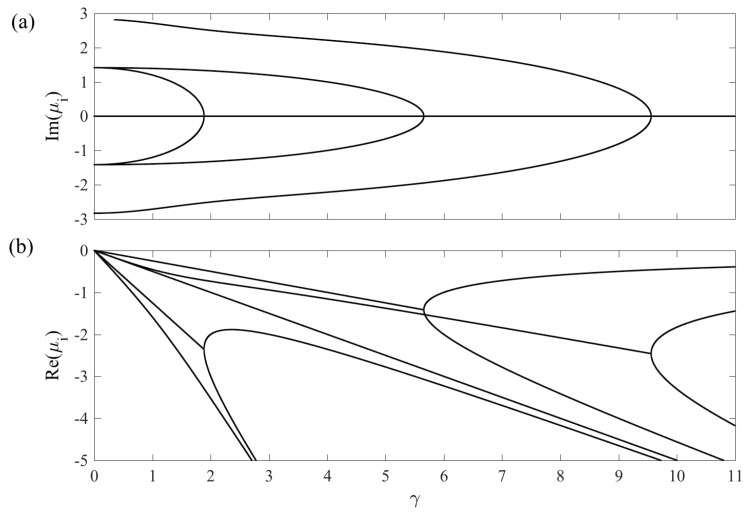
The imaginary (**a**) and real (**b**) parts of the eigenvalues for the Raman Λ system and for case (*a*); dephasing of the lower states |1〉 and |3〉. As long as γ≠0, all eight eigenvalues possess a negative real part implying that the Bloch vector decays to the origin. The EPs occur when the imaginary part vanishes (happening in pairs) and the real parts display a bifurcation-like behaviour. The dimensionless parameters are $G_{1}=G_{2}=1$ and Δ=0.

**Figure 5:**
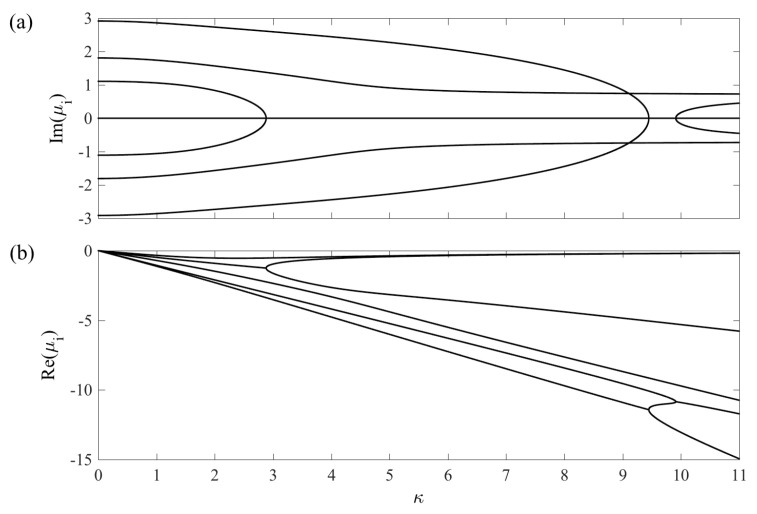
The imaginary (**a**) and real (**b**) parts of the eigenvalues for the Raman Λ system and for case (*b*); spontaneous emission of the excited state |2〉. A similar structure is found as for case (*a*), but with one new feature, namely the appearance of non-zero imaginary parts (seen around κ≈10). Even though the Liouvillian gap $\tilde{\Delta }\neq 0$ whenever κ≠0, it is possible to find a non-trivial steady state thanks to the non-vanishing pump term b (see the Appendix C). Note that both in this figure and in Figure 4, we consider rather large values of decay rates in order to demonstrate the general properties.

In Figure 4 and Figure 5, we display the real and imaginary parts of the eigenvalues of the Liouvillian matrices for case (*a*) and (*b*), respectively. In both cases, we have that the Liouvillian gap $\tilde{\Delta }$ is non-zero whenever the coupling to the environment is present. For case (*a*), where b=0, as we already mentioned this results in that the steady state is the maximally mixed state with R=0. For case (*b*), the real parts of the eigenvalues are also all non-positive, but as we pointed out the non-vanishing pump makes it possible that the steady state contains quantum coherences. The complex conjugation pairing of the eigenvalues is evident in the plots of the imaginary parts. The disappearance of imaginary parts at the EPs implies a splitting of the real parts, reminiscent of a bifurcation. At these points, the eigenvectors become real, which, however, is not enough to warrant that they represent physical states. In order for them to serve as physical states, their lengths must be relatively short. Indeed, given that the Bloch vector is real, it is always possible to construct a physical state $\hat{\rho }$ from it given that one shrinks its length sufficiently much [29]. In case (*a*), for large enough γs, all eigenstates become purely real after the three EPs. In case (*b*), however, a “reversed” EP takes place where two purely real eigenstates become imaginary upon increasing κ.

**Figure 6:**
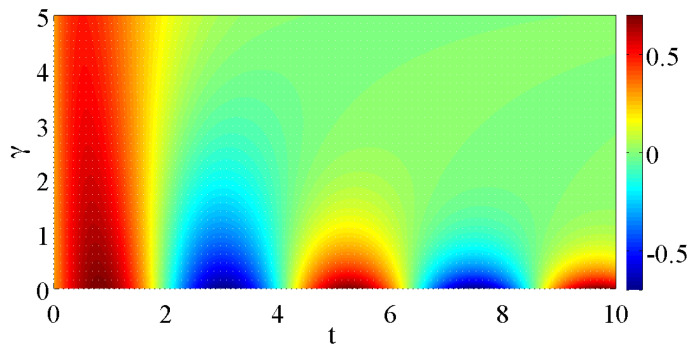
Population imbalance between the two lower states of a static Raman model under influence of dephasing, i.e., case (*a*), as a function of time and the system–environment coupling γ for an initial random pure state. For small γ, the evolution at this scale is predominantly unitary with clear Rabi oscillations between the two states, while, for larger γ, the decay of the imbalance is approximately exponential. The parameters are as in the previous figures, $G_{1}=G_{2}=1$ and Δ=0.

The EPs apparent in both spectra, Figure 4 and Figure 5, clearly demonstrate an example of non-analytic behaviour. A natural question arises whether their presence can result in visible effects in the system evolution, and in particular non-analytical behaviours. Indeed, the connection between EP’s and non-equilibrium phase transitions has been discussed in the past [30,31]. One may envision signatures of these in our model, for example the time-evolution of some initial state could qualitatively change if one varies γ or κ; whenever a splitting of the real parts occurs, the exponents rendering the decay change that could alter the system’s evolution. We explore this by calculating the population imbalance $Z=\mathrm{Tr}\left[(|1\rangle \langle 1|-|3\rangle \langle 3|)\hat{\rho }\left(t\right)\right]$ for an initial random pure state. The result for case (*a*) is shown in Figure 6. For small γ (<$G_{1},\;G_{2}$) and sufficiently short times, the evolution is dominated by unitary time-evolution, i.e., the population imbalance displays Rabi oscillations between the two states. When γ is increased, the inverse $\gamma^{-1}$ determines the fast time-scale and the relaxation occurs before any Rabi oscillations have time to manifest. The presence of non-analytic features of the eigenvalues are, however, not reflected in the time-evolution of the physical states of Figure 6.

It can be shown, however, that, for non-physical states, corresponding to complex eigenstates $R_{i}\left(t\right)$ non-analytic time-evolution is found upon quenching through an EP by considering a time-dependent γ(t) [23]. In this respect, we think of the instantaneous eigenstates $R_{i}\left(t\right)$ as the adiabatic states of M(t), and at an EP the characteristic time-scale diverges such that adiabaticity necessarily breaks down [32].

### 3.2. Dynamics—Numerical Results

The previous subsection analysed general effects stemming from the environment for a frozen Raman Hamiltonian, and not the actual interplay between external influence and the inherent STIRAP dynamics. This is the main focus of the present subsection.

If the Liouvillian gap $\tilde{\Delta }$ is large in comparison to the inverse time that sets the inherent time scale, Hamiltonian adiabatic evolution is not guaranteed by a large adiabaticity parameter *a*. Generally speaking, a large parameter *a* favours internal adiabatic evolution, but it implies an extended coupling to the environment, which, in return, tends to take the system out of its instantaneous adiabatic Hamiltonian eigenstate [23,24]. Consequently, one expects that there is an optimal $a_{opt}$ such that the intrinsic unitary evolution is close to adiabatic and at the same time excitations due to the environment are not too definite. This scenario should apply to case (*a*) representing dephasing, while for case (*b*) there should not be a trade-off between the two processes, i.e., a slow passage does not automatically lead to environment induced excitations since the state remains in desired adiabatic state. Recently, a similar scenario was also discussed in quantum many-body systems [33]. However, the parameter dependence of the optimal time $a_{opt}$ is not known. It is especially interesting to compare the optimal $a_{opt}$ with the rate γ. Note from Figure 4 that, for γ<2, the real parts of the eigenvalues $\mu_{i}$ scale linearly with γ, meaning that there is a linear dependence between the characteristic relaxation time scale and the inverse of the decay rate. It is reasonable to assume that $a_{opt}$ thereby must decrease for growing γ. We will see that this is only partly true, namely for small γ.

**Figure 7:**
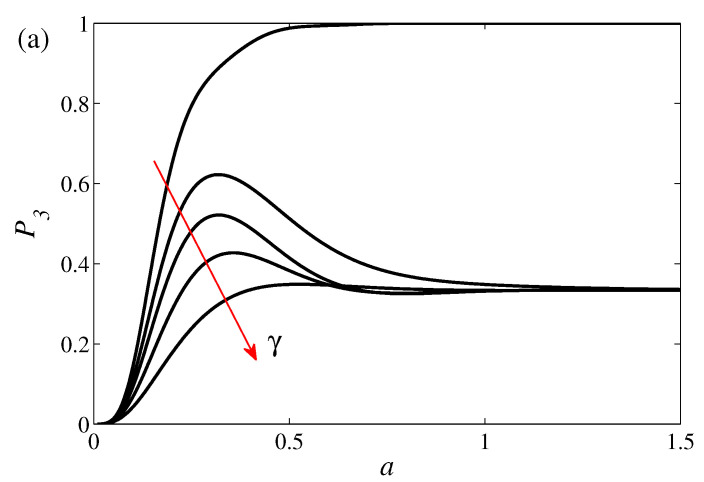

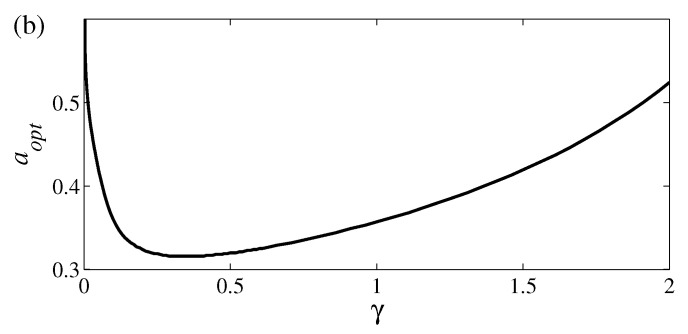
Upper plot (**a**): final population of the target state |3〉 in the case (*a*) for different adiabaticity parameters *a* and five different loss rates γ=0,1/4,1/2,1,2 in growing order with the arrow. In the closed case, upper curve for γ=0, the population is a monotonously increasing function of *a* demonstrating that adiabaticity is increased with a large *a*. As explained in the main text, for an open STIRAP process, the lower curves, there is an optimal $a_{opt}$ that maximises the population transfer. The evolution behaviour can be divided into two regimes; for small decay rates, one should chose *a* such that there is a balance between non-adiabatic excitations and environment induced excitations, while, for large γs, a Zeno-type effect sets in which implies that it might be favorable to prolong the process in order to achieve a more complete Zeno-freezing of the population transfer. The lower plot (**b**) displays this optimal adiabaticity parameter as a function of the loss rate γ. The two regimes are separated by the minima of the curve, and, to the left, we have $\gamma^{-1}$ dominated dependence and to the right a linear γ dependence. The remaining dimensionless parameters are $g_{0}=1$, Δ=0, and τ=σ=10.

The numerical results for the full time-dependent STIRAP problem for case (*a*) are displayed in Figure 7. As a measure of the efficiency of STIRAP, we calculate the population

(17) $$P_{i}=\langle i|\hat{\rho }\left(t_{f}\right)|i\rangle ,\;i=1,\;2,\;3,$$

at the final time $t_{f}$. For perfect transfer, all population ends up in the state |3〉, i.e., $P_{3}=1$. The integration interval $t\in [t_{i},t_{f}]$ ($t_{f}=-t_{i}=100$) is chosen long enough such that convergence has been reached. The uppermost curve in plot (a) represents the closed STIRAP and we find that *a* serves as an adiabaticity parameter, i.e., there is a one-to-one relation between its value and the success rate of the STIRAP. In the other examples displayed in the figure, γ≠0 and the maximum population transfer is not found for a→∞, but, for a finite *a*, which defines $a_{opt}$. For the decay rates used in the figure, the convergence to $P_{3}=1/3$ occurs for moderate *a* values. In fact, for any non-zero γ, we have that $P_{1}=P_{2}=P_{3}=1/3$ in the limit of large *a*. In this limit, the state is the maximally mixed one $\hat{\rho }=I/3$, which can be understood since it is also the instantaneous steady state of the corresponding Liouvillian. Nevertheless, when $G_{1}=G_{2}=0,$ any diagonal density matrix, in the diabatic basis, is a steady state, and hence, since the STIRAP pulses go to zero for large times, one could expect an asymptotic state different from the maximally mixed one. However, for large *a*s, the pulses are “sufficiently” non-zero to warrant relaxation towards the steady state ${\hat{\rho }}_{ss}=I/3$. The γ-dependence of the optimal $a_{opt}$ is shown in Figure 7b. For larger values of γ beyond 2, there is no longer a clear maximum any longer. The plot clearly indicates the divergence for γ→0, and as γ becomes nonzero we see a decrease in $a_{opt}$ as expected—the processess should not be too slow since then the environmental fluctuations hinder perfect transfer. In this regime, we find $a_{opt}\tau ∼\gamma^{-1},$ which is expected as it says that the STIRAP time-scale should be of the same order as the relaxation time-scale. However, for larger γ, beyond 0.4, the optimal time-scale starts to grow again! One roughly finds

(18) $$a_{opt}\tau \approx c_{0}+\frac{1}{\gamma }+c_{1}\gamma ,$$

for some constants $c_{0}$ and $c_{1}$ (that depend on the remaining system parameters). The linear γ-dependence setting in for larger decay rates is not intuitive. This suggests a slower STIRAP process when the coupling to the environment is getting larger. Of course, the mechanism behind this must be different from the one explained above leading to a $\gamma^{-1}$-dependence. If we study the actual form of the Liouvillian matrix (A7), we see that a non-zero γ has the effect of decreasing all the Bloch vector components $r_{i}$ apart from $r_{3}$ and $r_{8}$ which represent the diagonal elements of the density operator. Thus, as expected, the dephasing diminish the of diagonal coherence terms. If this reduction is definite, the dynamics enters into the Zeno regime—the absence of coherences block the population transfer. It seems that the explanation to this unintuitive result is a Zeno effect. For large γ, it might be favorable to consider slow processes in order to prohibit further population transfer, which would lower the final population of the desired state. We should point out, however, that this regime occurs for decay rates that are on the same order as the couplings $g_{0}$, and one may question the justification of describing the system by a Markovian Lindblad master Equation (1). Experimentally, it might be possible to simulate this regime and remain Markovian by utilizing so called engineered dissipation [34], which means that the Lindblad jump operators are monitored by the experimenter by cleverly couple stable states to dissipative ones.

**Figure 8:**
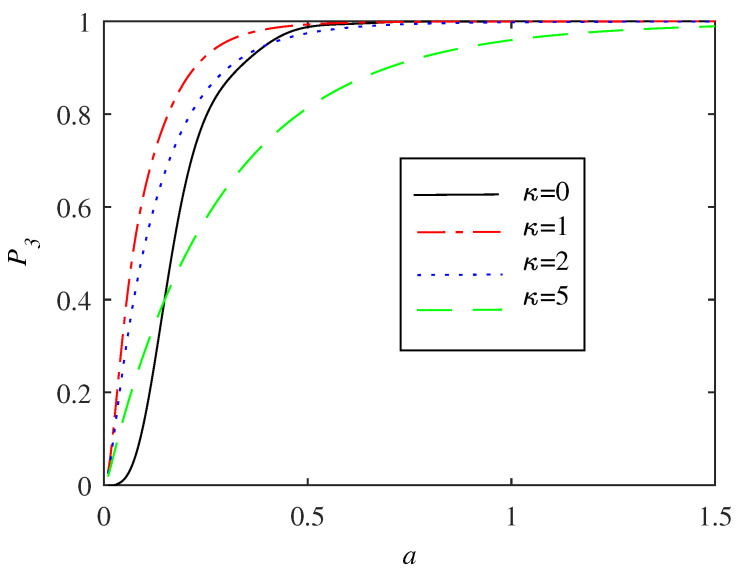
The same as the previous Figure 7a but for case (*b*) describing spontaneous emission of the intermediate |2〉 level. As was argued in the main text, since the adiabatic state is an instantaneous steady state for slow processes, i.e., large *a*s, the system state is transparent to the environment. For fast processes, when adiabaticity breaks down, it is found that the environment actually increases the population transfer, which can be ascribed the non-vanishing Liouvillian gap. However, there is a trade-off, a too strong coupling to the bath may lower the success rate as seen from the green curve. Indeed, in the limit of a very large γ, we recover the Zeno effect of Ref. [11] which manifest as the system frozen in the initial state |1〉.

Case (*b*) is conceptually different from case (*a*). The STIRAP adiabatic state $|\varphi_{0}\left(t\right)\rangle$ is a zero eigenvalue eigenstate of the Lindblad jump operators ${\hat{L}}_{1,2}$ of Equation (12), meaning that it is an instantaneous steady state and moreover transparent to the environment. Perfect population transfer is thereby expected in the deep adiabatic regime. This is also found numerically, as demonstrated in Figure 8. Contrary to the case of dephasing, here the population of the target state is always a monotonously increasing function of *a* regardless of the value of κ, or, put in other words, *a* is a proper parameter to characterise efficiency of the process.

In principle, adiabaticity and efficiency need not be equivalent for open systems. In fact, adiabaticity can be introduced in various ways for open quantum systems. This issue has been raised in a series of papers [23,24,35,36]. One definition is to define it by saying that the population transfer between different adiabatic states of M vanishes (A4). At an EP, adiabaticity is then doomed to break down. Following this, the adiabatic criterium (9) can be modified to open systems in a rather direct way [23]. As is clear, the openness may greatly affect the criteria and typically the environment induces additional excitations [37,38]. In this respect, something interesting occurs for small *a* when the STIRAP is not fully adiabatic; the spontaneous emission increases the efficiency of the population transfer. This derives from the presence of a Liouvillian gap $\tilde{\Delta }$, which implies that the adiabatic state $|\varphi_{0}\left(t\right)\rangle$ is partly protected from non-adiabatic excitations. One way of seeing it is that a non-zero κ helps the process to become more adiabatic [35]. Thus, contrary to the standard situation where the fluctuations from the environment induces excitations, in the non-adiabatic regime, the environment prevents the system from taking it out from the instantaneous Hamiltonian adiabatic state. The phenomenon is related, but still different from the Zeno-effect discussed in Ref. [11]. There, in the overdamped regime, the environment prohibits the system from leaving the initial state |1〉. In the present situation, we are far from overdamped (except the dashed green line of Figure 8) and the environment induced relaxation takes you back onto the adiabatic state and not onto the initial state. Remember that the adiabatic state is also an instantaneous steady state and as soon as the Hamiltonian drives you out of this state, the environment pushes you back towards it. It should be clear that the same mechanism does not work for case (*a*) since the desired adiabatic state is not an instantaneous steady state of the full model. There, we found instead a different manifestation of the Zeno physics, namely a freezing of population transfer between the different diabatic states.

As a final remark, the jump operators (12) describe the decay into an incoherent mixture of |1〉 and |3〉. One may ask whether a “coherent” decay [39], like, for example, as represented by the Lindblad jump operator $\hat{L}=\left|1\rangle \langle 2\right|+\left|3\rangle \langle 2\right|$, would affect the result of Figure 8. We tried for a couple of different such jump operators and found that the coherence does not play a quantitative role; any such decay protects the adiabatic state during fast processes.

## 4. Conclusions

Starting from the Liouvillian, we have analysed the open STIRAP problem. The situations of either dephasing or dissipation were considered separately and in both cases we found surprising results. An important difference between the cases is that for dissipation the Lindblad jump operator is Hermitian and one consequence is that, for the generalized Bloch Equation (14), one inevitably has a non-vanishing pump term b [32]. This, in return, implies that the steady state is non-trivial, in contrast to the case of dephasing where the instantaneous steady state is the maximally mixed one. In particular, the desired adiabatic state is also the instantaneous steady state for the case of dissipation, and the slower the process becomes, the more efficient population transfer to the target state. This is in agreement with the definition of adiabaticity for open quantum systems in terms of steady states [35], which states that, if the system is initially in a steady state, and the process is infinitely slow, then the system remains in the same instantaneous steady state for all times. For dissipation, the interesting new result is found for processes that are not perfectly adiabatic, i.e., during the evolution, non-adiabatic excitations take the system out from the instantaneous steady state. Here, it is found that the environment can “protect” the steady state in the sense that excitations out of it are counteracted by forcing the state to relax back to the instantaneous steady state. This is a sort of Zeno effect, and the system is locked to be in the adiabatic state.

In the situation of dephasing, we found that the evolution can be separated into two regimes depending on the strength of the decay rate γ. For weak γ, the environment indeed causes fluctuations that decrease the transfer efficiency, and one should therefore not make the process too slow. Instead, one should choose it as a trade-off between minimizing both the Hamiltonian non-adiabatic excitations and the environmental induced excitations. This result has been mentioned in the past [9,32,33,40], but here we suggest that the optimal time-scale for the process is of the same order as the inverse decay rate. What was surprising, however, was that, for larger decay rates γ, the numerics showed that a slower process might actually be advantageous. This is in conflict with the general knowledge that the environment will induce additional unwanted excitations. We argued that this is again a result of a Zeno-type effect. If the process is slow, all coherence terms of the state are quenched and the population transfer between the diabatic states is considerably slowed down. Of course, the Zeno freezing should not be perfect early on, since then there will be no population transfer at all. Instead, the “freezing” should occur at the time when the population in the target state is the largest.

To the best of our knowledge, both of these Zeno effects are new. In particular, they are different from the Zeno effect analyzed in Ref. [11]. In that work, the dynamics was completely frozen by the strong coupling to the environment, while, in our work, for dissipation, the dynamics are only partly frozen; the system is still evolving, but it follows an adiabatic state. Thus, the Zeno freezing we consider is not perfect in the sense that evolution still occurs.

An experimental system that could be suitable for exploring our theoretical predictions is trapped ions [41], where STIRAP is a standard tool for state preparation (see, for example, [42]). In these systems, both the initialisation and detection is almost perfect. By engineering the dissipation/decoherence, which has also been demonstrated experimentally [34], one should be able to construct the desired jump operators and the corresponding decay rates γ and κ.

## Abbreviations

The following abbreviations are used in this manuscript:

STIRAP	Stimulated Raman Adiabatic Passage
AMO	Atomic, Molecular and Optical
EP	Exceptional Point

## Appendix A. Gell–Mann Matrices

For the three-level STIRAP problem, rewriting the density operator on the Bloch form implies expanding it in the identity plus the regular Gell–Mann matrices [43]. The Gell–Mann matrices

(A1) $$\begin{array}{cc} \lambda_{1}=\left[\begin{array}{ccc} 0 & 1 & 0\\ 1 & 0 & 0\\ 0 & 0 & 0 \end{array}\right], & \lambda_{2}=\left[\begin{array}{ccc} 0 & -i & 0\\ i & 0 & 0\\ 0 & 0 & 0 \end{array}\right],\\ \lambda_{3}=\left[\begin{array}{ccc} 1 & 0 & 0\\ 0 & -1 & 0\\ 0 & 0 & 0 \end{array}\right], & \lambda_{4}=\left[\begin{array}{ccc} 0 & 0 & 1\\ 0 & 0 & 0\\ 1 & 0 & 0 \end{array}\right],\\ \lambda_{5}=\left[\begin{array}{ccc} 0 & 0 & -i\\ 0 & 0 & 0\\ i & 0 & 0 \end{array}\right], & \lambda_{6}=\left[\begin{array}{ccc} 0 & 0 & 0\\ 0 & 0 & 1\\ 0 & 1 & 0 \end{array}\right],\\ \lambda_{7}=\left[\begin{array}{ccc} 0 & 0 & 0\\ 0 & 0 & -i\\ 0 & i & 0 \end{array}\right], & \lambda_{8}=\frac{1}{\sqrt{3}}\left[\begin{array}{ccc} 1 & 0 & 0\\ 0 & 1 & 0\\ 0 & 0 & -2 \end{array}\right], \end{array}$$

(as their generalisations to higher dimensions) are orthogonal in the sense that $\mathrm{Tr}\left[\lambda_{i}\lambda_{j}\right]=2\delta_{ij}$, and, furthermore, they are traceless. The commutation relations are

(A2) $$\left[\lambda_{i},\lambda_{j}\right]=i2f^{ijk}\lambda_{k},$$

with the antisymmetric tensor $f^{ijk}$ obeying

(A3) $$\begin{array}{c} f^{123}=1,\\ f^{147}=f^{165}=f^{246}=f^{257}=f^{345}=f^{376}=\frac{1}{2},\\ f^{458}=f^{678}=\frac{\sqrt{3}}{2}. \end{array}$$

Any remaining elements, for example with two or three identical superscripts equal, are identically zero.

## Appendix B. Some General Properties of the Liuvillian

The Liouvillian matrix M is not Hermitian, meaning that its eigenvalues may be complex and furthermore one has to separate between left and right eigenvectors. Even though the left and right eigenvectors may be different, there always exists a similarity matrix S such that

(A4) $$D=SMS^{-1},$$

where the matrix D is on Jordan–Block form [44]. That is, each block is comprised of degenerate eigenvalues on its diagonal and ones on the superdiagonal. Blocks of dimension higher than one appear at the EPs. We note that, in the special situation of a closed system, the eigenvectors of M corresponds to the states ${\hat{\rho }}_{ij}=\left|i\rangle \langle j\right|$, where |i〉 is the *i*’th eigenstate of the Hamiltonian. Another property of the Liouvillian matrix is that it is real and thereby its trace is also real, which has the consequence that its eigenvalues can be grouped in pairs with opposite imaginary parts.

There is a further important observation to be stressed about the Bloch representation of the master equation. For any physical state $\hat{\rho }$, we must have that it is: (i) Hermitian; (ii) positive semi-definite; and (iii) normalised with unit trace. For qubits, i.e., D=2, all states with 0≤R≤1 are physical, implying that the Bloch sphere comprises all the allowed states. In higher dimensions, for qutrits and, more generally, qudits, only part of the Bloch sphere represents actual physical states [20,29]. We mentioned above that the eigenstates of the master equation for a closed system are ${\hat{\rho }}_{ij}=\left|i\rangle \langle j\right|$, meaning that there are *D* times as many eigenstates for the Liouvillian matrix M than to the Hamiltonian. These additional states are, of course, those with i≠j that are traceless and hence do not represent physical states. Note further that ${\hat{\rho }}_{ij}=\left|i\rangle \langle j\right|$ gives $D^{2}$ states, but the dimension of M is $D^{2}-1$. This extra state, missing in our Bloch representation, appears since the ${\hat{\rho }}_{ii}$ states are not linearly independent (we have $\sum_{i=1}^{D}{\hat{\rho }}_{ii}=I$) and one of them is always redundant. For non-zero coupling to the environment, one typically finds most states unphysical, and interestingly the number of physical states may not be the same as for the closed case. A direct requirement for having a physical state is that the corresponding eigenvalue of M is real (otherwise R is complex and $\hat{\rho }$ not Hermitian). We have found for our three-level problem that whenever we have a real R, the corresponding state $\hat{\rho }$ is physical provided that *R* is sufficiently small, i.e., there is a ball surrounding the origin R=0 containing only physical states. As a result, for the open STIRAP problem, we need a minimum of two real eigenvalues (It can be shown that the real part of an eigenvalue of M has to be nonpositive [28].) of M (remember that they are grouped in pairs) and the corresponding states will be physical. It is intriguing that the unphysical states may show interesting properties that are missing for the physical ones. In Ref. [45], the geometric phases of these were studied, and it was shown that this phase is normally non-vanishing, while for the physical states the geometric phase strictly vanishes. Geometric phases connected to encircling EPs have been explored as well [46], but the link between the phases found for non-physical states and those related to EP’s were not studied in Ref. [45].

While unphysical, these states are still crucial for the time-evolution [26]. If b=0, and we denote the right eigenvectors $R^{\left(i\right)}$, we have that the time evolved Bloch vector can be expressed as

(A5) $$R\left(t\right)=\sum_{i=1}^{8}c_{i}e^{\mu_{i}t}R^{\left(i\right)},$$

where $\mu_{i}$ are the eigenvalues of the Liouvillian, $c_{i}$ are the coefficients determined by the initial state R(0), and we have assumed a time-independent Liouvillian matrix M. Even though the right (left) eigenvectors are not orthogonal in general, the series expansion is unique given that the vectors are normalized and we do not “sit” on an EP. The sum will in general include unphysical Bloch vectors, which does not imply that the full sum R(t) is also unphysical. Indeed, it has to be as long as R(0) is physical. Note that we must have Re(μi)≤0 [28]. If $\mu_{i}=0$, the corresponding Bloch vector $R^{\left(i\right)}$ is stationary and if the state ${\hat{\rho }}^{\left(i\right)}$ is a steady state of the master equation. Whenever the eigenvalues contain real parts, these will cause an exponential decay of the corresponding terms in the sum. If the Lindblad jump operators ${\hat{L}}_{i}$ are Hermitian, the maximally mixed state $\hat{\rho }=I/D$ is a steady state, and, if this is also the unique steady state, we must have that all eigenvalues have non-zero real parts [47].

For b≠0, which typically occurs for non-hermetian Lindblad jump operators ${\hat{L}}_{i}$, the situation is more complicated. Here, a stationary state is represented by a Bloch vector $R=-M^{-1}b$ given that M is invertible. If M is not invertible, however, the system is underdetermined and you find a connected convex manifold of solutions. Introducing the matrices V and U such that $E=V^{t}\mathrm{MU}$ with E diagonal, the right eigenvectors of M evolve in time as

(A6) $$R^{\left(i\right)}\left(t\right)=R^{\left(i\right)}\left(0\right)e^{\mu_{i}t}+\left(e^{\mu_{i}t}-1\right)V^{t}b/\mu_{i}.$$

For b=0, we recover the exponential time-dependence of Equation (A5). We see, as stated above, that for b≠0 a negative real part of $\mu_{i}$ is not sufficient to warrant that the steady state is the simple maximally mixed one as it is for Hermitian Lindblad jump operators. In these situations, looking at the dynamical Equation (14), it is clear that the b term acts as a sort of “pump” which has the effect of restoring some coherence/purity of the state $\hat{\rho }\left(t\right)$.

It might well happen that M is not diagonalisable. This happens at the EPs in the parameter space [12,13]. At an EP, the real parts of two eigenvalues, $\varepsilon_{1}\left(\nu \right)$ and $\varepsilon_{2}\left(\nu \right)$ (with ν some parameter), coalesce and at this “degeneracy point” the corresponding eigenstates become identical, in stark contrast to a degeneracy of an Hermitian operator where the eigenstates can always be chosen orthogonal. The EP is also characterised by a square-root singularity [28], and upon encircling such a singularity once, the corresponding eigenstates are interchanged, while encircling it twice gives you back the same state up to an overall sign change [46,48]. This generalises the geometric phase appearing when enclosing a *Dirac point* in condensed matter physics or a *conical intersection* in molecular/chemical physics [49] to degeneracy points for non-Hermitian operators.

## Appendix C. STIRAP Liouvillian Matrices

Employing the commutation properties of the Gell–Mann matrices (A2), the derivation of the Liouvillian matrix and pump is straightforward. In the case (*a*), representing pure dephasing (see Equation (11)), the pump term vanishes. The matrix is found to be

(A7) $$M\;=\;\;\left[\;\;\begin{array}{cccccccc} \;-\gamma \;/2 & \Delta & 0 & 0 & G_{2} & 0 & 0 & 0\\ -\Delta & \;-\gamma \;/2 & \;-2G_{1} & \;-G_{2} & 0 & 0 & 0 & 0\\ 0 & 2G_{1} & 0 & 0 & 0 & 0 & -G_{2} & 0\\ 0 & G_{2} & 0 & -2\gamma & 0 & 0 & -G_{2} & 0\\ -G_{2} & 0 & 0 & 0 & -2\gamma & G_{1} & 0 & 0\\ 0 & 0 & 0 & 0 & \;-G_{1} & \;-\gamma \;/2 & -\Delta & 0\\ 0 & 0 & G_{2} & G_{1} & 0 & \Delta & -\gamma \;/2 & \;-\sqrt{3}G_{2}\\ 0 & 0 & 0 & 0 & 0 & 0 & \;\sqrt{3}G_{2} & 0 \end{array}\;\;\;\right]\;\;.$$

Note that M is skew-symmetric and real. In odd dimensions, the skew-symmetric property implies that the matrix is singular, but this is not the case here as the dimension is eight. The skew-symmetry property also implies that the eigenvalues are paired as pointed out in the main text. Another observation is that the number of elements proportional to $G_{2}$ is higher than those proportional to $G_{1}$, which might seem spurious at first. The reason for this lies in the definition of the Gell–Mann matrices (A1); there is an asymmetry between the different SU(2) subgroups of the SU(3) group and the atomic transitions are connected to two different subgroups.

For the second case (*b*) of a dissipating excited state |2〉 (see Equation (12)), the jump operators are no longer Hermitian and we indeed find a non-zero pump contribution,

(A8) $$M\;=\;\;\left[\;\;\begin{array}{cccccccc} \;-\gamma \;/2 & \Delta & 0 & 0 & G_{2} & 0 & 0 & 0\\ -\Delta & \;-\gamma \;/2 & \;-2G_{1} & -G_{2} & 0 & 0 & 0 & 0\\ 0 & 2G_{1} & -\gamma & 0 & 0 & 0 & -G_{2} & \gamma \;/\;\sqrt{3}\\ 0 & G_{2} & 0 & -\gamma \;/2 & 0 & 0 & -G_{2} & 0\\ -G_{2} & 0 & 0 & 0 & \;-\gamma \;/2 & G_{1} & 0 & 0\\ 0 & 0 & 0 & 0 & -G_{1} & \;-\gamma & -\Delta & 0\\ 0 & 0 & G_{2} & G_{1} & 0 & \Delta & -\gamma & \;-\sqrt{3}G_{2}\\ 0 & 0 & 0 & 0 & 0 & 0 & \;\sqrt{3}G_{2} & 0 \end{array}\;\;\;\right]\;\;,$$

and

(A9) $$b^{t}=\left[\begin{array}{cccccccc} 0 & 0 & \gamma /\sqrt{3} & 0 & 0 & 0 & 0 & 0 \end{array}\right].$$

Contrary to the first case, the matrix is no longer skew-symmetric due to a non-zero term $M_{38}=\gamma /\sqrt{3}$. It is clear that this contribution is connected to the non-vanishing pump b that has also a non-zero term as its third element.
